# The pandemic generation: Investigating the long‐term impact of a large‐scale stressor on the anxiety of children

**DOI:** 10.1002/jcv2.70105

**Published:** 2026-02-25

**Authors:** Michiel A. J. Luijten, Hedy A. van Oers, Jacintha M. Tieskens, Marlene A. Werner, Josjan Zijlmans, Lotte Haverman, Tinca J. C. Polderman, Lianne P. de Vries

**Affiliations:** ^1^ Child and Adolescent Psychiatry and Psychosocial Care Amsterdam University Medical Center Location University of Amsterdam Emma Children's Hospital Amsterdam The Netherlands; ^2^ Amsterdam Public Health Amsterdam University Medical Center Mental Health Amsterdam The Netherlands; ^3^ Epidemiology and Data Science Amsterdam University Medical Center Vrije Universiteit Amsterdam Amsterdam The Netherlands; ^4^ Amsterdam Public Health Amsterdam University Medical Center Methodology Amsterdam The Netherlands; ^5^ Amsterdam Reproduction and Development Child Development Amsterdam The Netherlands; ^6^ Leiden University Medical Center Curium Leiden The Netherlands; ^7^ Amsterdam University Medical Center Location University of Amsterdam, Sexology and Psychosomatic Gynaecology Amsterdam The Netherlands; ^8^ Cancer Center Amsterdam Amsterdam The Netherlands; ^9^ Department of Child and Adolescent Psychiatry and Psychosocial Care Amsterdam University Medical Center Location Vrije Universiteit Amsterdam Amsterdam The Netherlands; ^10^ Levvel Academic Center for Child and Adolescent Psychiatry Amsterdam The Netherlands; ^11^ Karakter Child and Adolescent Psychiatry University Centre Nijmegen The Netherlands; ^12^ Accare Child Study Center Groningen The Netherlands; ^13^ Department of Biological Psychology Vrije Universiteit Amsterdam Amsterdam the Netherlands

**Keywords:** anxiety, cluster, COVID‐19, mental health, trajectories

## Abstract

**Background:**

As a large‐scale global stressor, the COVID‐19 pandemic has a substantial impact on the mental health of children and adolescents. In this longitudinal study we investigate long‐term trajectories of anxiety of children during and after the pandemic (April 2020–April 2023), and identify associated risk factors.

**Methods:**

A total of 514 children from a representative sample of the Dutch general population completed the Patient Reported Outcomes Measurement Information System Anxiety computerized adaptive test at least four of seven bi‐annual assessments (from April 2020 to April 2023). Longitudinal k‐means clustering analyses were conducted to identify distinct anxiety trajectories. The optimal solution was selected based on the Calinski‐Harabasz criterion and interpretability. We assigned participants to their best fitting cluster and compared sociodemographic characteristics between the clusters using *T*‐tests and chi‐square tests to identify risk factors.

**Results:**

We identified five distinct longitudinal trajectories of anxiety; stable high (*n* = 71, 13.8%), stable medium (*n* = 109, 21.2%), stable low (*n* = 147, 28.6%), recovering (*n* = 100, 19.5%) and delayed increase trajectories (*n* = 87, 16.9%). The recovering group showed a return to anxiety levels within normal limits over time, while the delayed increase group experienced increased anxiety symptoms after the pandemic. Loss of income from the parents was identified as an important risk factor of belonging to the stable high trajectory (OR range 4.1–8.2, *p* < 0.001).

**Conclusion:**

Children followed distinct anxiety trajectories during and after the COVID‐19 pandemic, showing variability in their long‐term mental health responses. Five trajectories were identified, and parental loss of income emerged as a strong risk factor for less favorable trajectories. These findings show the importance of monitoring mental health over longer periods and especially children that experience additional adversity on top of global stressors might be at risk for lasting mental health problems.

## Key Points

**What's known?**

- The COVID‐19 pandemic had a large impact on the mental health of children. Previous studies have shown that being faced with a pandemic outbreak and the subsequent regulations have increased anxiety levels in children.

**What's new?**

- We assessed the impact of returning to normal life (e.g., post‐pandemic assessment) on anxiety.
- We identified five distinct trajectories of anxiety over a period of April 2020 to April 2023. Some children recovered and others remained stable throughout the pandemic.

**What's relevant?**

- Our findings suggest that a non‐negligible group of individuals reported worse anxiety after the pandemic regulations ended and society returned to normal.
- Loss of income of the parents is associated with higher levels of anxiety in their children, suggesting that extra parental stress plays a role in the development and maintenance of anxiety problems.

## INTRODUCTION

Mental health problems during childhood, adolescence, and young adulthood have a substantial impact on daily life and later development and lead to increased risk of psychopathology later in life (Clayborne et al., 2019; Patel et al., 2007). Recent estimates indicate that around 14% of adolescents are diagnosed with a mental disorder, and up to 34% report significant symptoms of depression or anxiety (Racine et al., 2021; Shorey et al., 2022). The COVID‐19 pandemic increased concerns about child and adolescent mental health, as the presence of the virus, lockdowns, school closures, and social isolation placed additional stress and limitations on children (Wolf & Schmitz, 2024).

Although previous studies have shown a general pattern of deteriorating mental health, notably anxiety and depression, across large cross‐sectional samples of children and adolescents (Luijten et al., 2021; Ravens‐Sieberer et al., 2022; van Oers et al., 2024), individual variability was often not taken into account. Yet, there is likely individual variability in the psychological responses to the pandemic and the recovery from the pandemic which cannot be detected by observing average responses only. Not every child has been affected the same way throughout the pandemic (Bruining et al., 2021); some children are less affected or have recovered quickly after the start of the pandemic, while others continue to struggle with symptoms of anxiety and depression. Several studies have investigated and identified risk factors for consistent mental health problems during the pandemic, which include age (older), sex (female), having a low family income or financial worries, parental stress, and being from a single‐parent family (Hafstad et al., 2021; Luijten et al., 2021; McArthur et al., 2023; Raw et al., 2021; Wolf & Schmitz, 2024).

However, less is known about how children's mental health develops differently from one child to another over time, during and after the pandemic, and what factors influence these differences. Using statistical techniques, such as latent class analysis or clustering, groups of individuals with similar patterns of mental health over time can be detected and identified. Several studies have investigated and identified longitudinal patterns of mental health in children and adolescents during the pandemic (up until 2022) revealing four distinct mental health trajectories: (1) consistently very good, (2) consistently good, (3) consistently poor and (4) a trajectory of recovering mental health (de Heer et al., 2025; Guzman Holst et al., 2023; Houghton et al., 2022; Kaman et al., 2024; van Loon et al., 2022). These studies have identified certain risk factors for having an unfavorable trajectory (consistently poor/deteriorating), such as migration background, being from a single‐parent family and less perceived social support (de Heer et al., 2025; van Loon et al., 2022). However, as data collection in these studies was limited to the early pandemic period (2020–2022), it remains unclear how children's mental health developed in the longer term, and whether these early risk factors continue to influence post‐pandemic mental health.

Longer‐term studies are needed to understand how children's mental health evolves beyond the pandemic and to identify children who are at risk for lasting mental health problems. In the Netherlands, governmental COVID‐19 regulations were lifted in the spring of 2022, marking a key transition point. In this study, we included post‐pandemic measurements (i.e., after governmental regulations ended) up to April 2023, with the goal of identifying patterns of resilience and vulnerability over time for anxiety in children. The primary aim of the study is to identify if distinct trajectories of anxiety symptoms in children throughout and after the pandemic exist. A secondary aim is to identify risk factors for these trajectories by examining associated sociodemographic characteristics that may predict trajectories of prolonged mental health problems.

## METHOD

### Participants & procedure

We collected data from a representative sample of children and adolescents from the general population through the Dutch panel agency PanelInzicht. We performed measurements bi‐annually from April 2020 until March 2023, resulting in a total of seven measurements. We tried to include as many repeated measures as possible by first approaching participants with previous measurements and subsequently adding new participants to reach a total sample size of 1000 children per measurement.

We used a two‐step stratified sampling procedure to obtain a representative sample, in which people were invited in two waves. The invitations for the second wave were adapted to the response rates of the first wave (e.g., if response rate was low for younger children in wave 1, more young children were invited in wave 2 to correct for this). The panel agency approached parents of participating children who then approached their child. Both parent and child provided informed consent and subsequently completed the questionnaires through a KLIK PROM (patient‐reported outcome measures) portal research website. Parents completed the sociodemographic questionnaire and children completed the PROMs. The data collection was approved by the Medical Ethics Testing Committee (METC) of the Amsterdam UMC. More detailed information on the data collection procedure, measurement occasions and representativeness of the data can be found in van Oers et al. (2024).

For this study, we selected participants that had completed at least four measurements occasions to be included in the longitudinal clustering analyses (*n* = 514). We checked whether the selected participants differed on sociodemographic characteristics compared to the full sample (see Table 1), using chi‐square tests of equal proportions for categorical variables and independent *t*‐tests for continuous variables. We considered a medium effect size (odds ratio >1.5 or <0.67 and Cohen's *D* > 0.5) to be a relevant difference between samples. The samples differed on the proportion of children with parents that have had a loss of income during the pandemic (see Table 1). The longitudinal sample contained a higher proportion (OR 1.83) of parents with a loss of income (24.3%) than the full sample (14.9%).

**TABLE 1:** Sociodemographic characteristics of the selected longitudinal sample and the full sample.

	Longitudinal sample (*N* = 514)	Full sample (*N* = 7781)	Effect size^a^
Sex; % females	51.2%	49.1%	1.08
Age (SD)	13.07 (2.94)	13.78 (3.14)	0.23
Single parent family; % yes	20.3%	20.3%	1.00
Loss of income; % yes	24.3%	14.9%	**1.83**
Urbanisation			
>2500 addresses per km^2^	15.7%	16.5%	0.94
>1500–2500 addresses per km^2^	27.1%	27.3%	0.99
>1000–1500 addresses per km^2^	20.2%	20.4%	0.99
>500–1000 addresses per km^2^	22.5%	21.5%	1.06
<500 addresses per km^2^	14.5%	14.3%	1.02
Education level parent^b^			
Practical vocational	8.1%	8.9%	0.90
Intermediate	45.8%	48.0%	0.91
Theoretical vocational	46.0%	43.0%	1.13
Parents born in the Netherlands^c^			
Neither	2.9%	3.2%	0.90
One	5.6%	7.6%	0.72
Both	87.0%	88.9%	0.83

^a^ Odds ratio for categorical variables, Cohen's *D* for continuous variables—bold represents a medium effect size or higher.

^b^ Practical vocational (primary education, lower vocational education, or lower and middle general secondary education), intermediate (middle vocational education, higher secondary education, or pre‐university education), or theoretical vocational (higher vocational education or university).

^c^ Percentages may not add up to a 100% as there are missing values.

### Measures

#### Sociodemographic questionnaire

The sociodemographic questionnaire was completed at each measurement occasion by parents and contained information on themselves and their child. Parents provided information on their age, sex, country of birth, number of children within the household, marital status, education level and postal code. Parental educational level was categorized into practical vocational (primary education, lower vocational education, or lower and middle general secondary education), intermediate (middle vocational education, higher secondary education, or pre‐university education), or theoretical vocational (higher vocational education or university). Postal codes were used to determine the degree of urbanisation of the household and were subsequently transformed into an ordinal variable with five categories ranging from no urbanisation (<500 addresses per km^2^) to very strong urbanisation (>2500 addresses per km^2^). In addition, parents were asked whether there was a loss of income due to any negative change in work situation (either in number of hours, income or employment status) due to the COVID‐19 regulations.

#### PROMIS pediatric anxiety item bank v2.0

Children self‐reported anxiety during each wave using the Patient Reported Outcomes Measurement Information System (PROMIS) Anxiety item bank v2.0. We administered the item bank as computerized adaptive test (CAT). CATs allow to tailor the questions to the level of anxiety of a child to obtain a reliable estimate in fewer questions (Cella et al., 2007). The PROMIS measure uses a 7‐day recall period, and items are scored on a five‐point Likert scale. All item response categories range from 1: “never” to 5: “(almost) always.” Total scores are calculated by transforming the item scores into a T‐score which has a mean of 50 and standard deviation (SD) of 10 in the U.S. general population. The PROMIS Anxiety item bank has been selected by the American Psychiatric Association as level‐2 assessment measure (i.e., suitable for monitoring and evaluating psychiatric disorders from the DSM‐5). The administration of PROMIS Anxiety as CAT has previously shown to be an efficient and reliable method of assessing anxiety in Dutch children and did not show any differential item functioning for age, making it suitable for longitudinal assessment (Klaufus et al., 2021). Pre‐pandemic reference values of the Dutch general population for the mean was 43.8 with cut‐off values for mild (50.8) and severe (61.5) anxiety (Klaufus et al., 2021).

### Statistical analysis

#### Clustering

We investigated longitudinal trajectories through k‐means clustering (Genolini & Falissard, 2011). K‐means clustering is a clustering technique that allows for more flexibility of the data as it does not require fulfillment of various assumptions on parameterisation and normality, while allowing the shape of trajectories to be flexible. There are drawbacks to k‐means clustering, which makes it less attractive, specifically for longitudinal data. The number of trajectories needs to be known beforehand and missing data may not be present for calculating certain quality criteria. Genolini & Falissard have made adjustments to improve k‐means clustering for longitudinal data and integrated it into the “KmL” package (Genolini & Falissard, 2011). In addition, k‐means clustering requires multiple iterations, otherwise it may converge to local minima.

The first step in this approach is to assess the optimal number of clusters. This is done by running multiple k‐means clustering analyses and calculating the Calinski‐Harabasz criterion (Caliński & and Harabasz, 1974) for each iteration. This criterion represents the within‐ and between cluster sum of squares. For calculating the criterion, the “KmL” package handles missing data by imputation. We used the recommended bisector linear interpolation to impute missing values (Genolini & Falissard, 2011). For the optimising the clustering procedure, we used the default, recommended, Euclidean distance with Gower adjustment (Genolini & Falissard, 2011). The Gower adjustment ensures that distances are not inflated when participants have missing measurement occasions.

We tested three to six clusters to maintain a parsimonious model and calculated the Calinski‐Harabasz criterion. For each number of clusters, several iterations were performed to obtain an optimized solution. Subsequently, we selected the best‐fitting model for each number of clusters and compared the criterion to select the optimal number of clusters. We interpreted the trajectories of this best fitting model and provide a trajectory description. For each trajectory we report mean Anxiety T‐scores (with confidence intervals (CI)) per timepoint. If CI do not overlap with the previous measurement, we interpret this as a significant change in anxiety levels. Trajectories in which there were no significant changes were considered stable trajectories, providing only limited information about the effects of the pandemic. We judged the clusters on interpretability and the presence of meaningful, informative trajectories, as it is common that clustering analysis maximize Euclidean distance through stable trajectories (Pierce et al., 2021; Rong et al., 2024). Therefore, if the optimal number of clusters contained only stable trajectories, we explored the next best‐fitting model until we could identify one or more unstable trajectories.

#### Risk factors

Each individual was classified according to their most likely trajectory. To identify possible risk factors for the different trajectories we subsequently tested clusters for differences in all available sociodemographic characteristics, which includes sex, age, being from a single‐parent family, degree of urbanization of the household, parental education level, parental country of birth and any loss of income of the parents during the pandemic. For continuous variables we performed an ANOVA with post‐hoc analyses to assess pairwise differences. For nominal variables we performed chi‐square tests of equal proportions through crosstabulation and report odds ratios (OR). Results for these tests were considered significant with a *p*‐value of 0.007, applying Bonferroni correction for multiple testing.

## RESULTS

### Clustering

**FIGURE 1:**
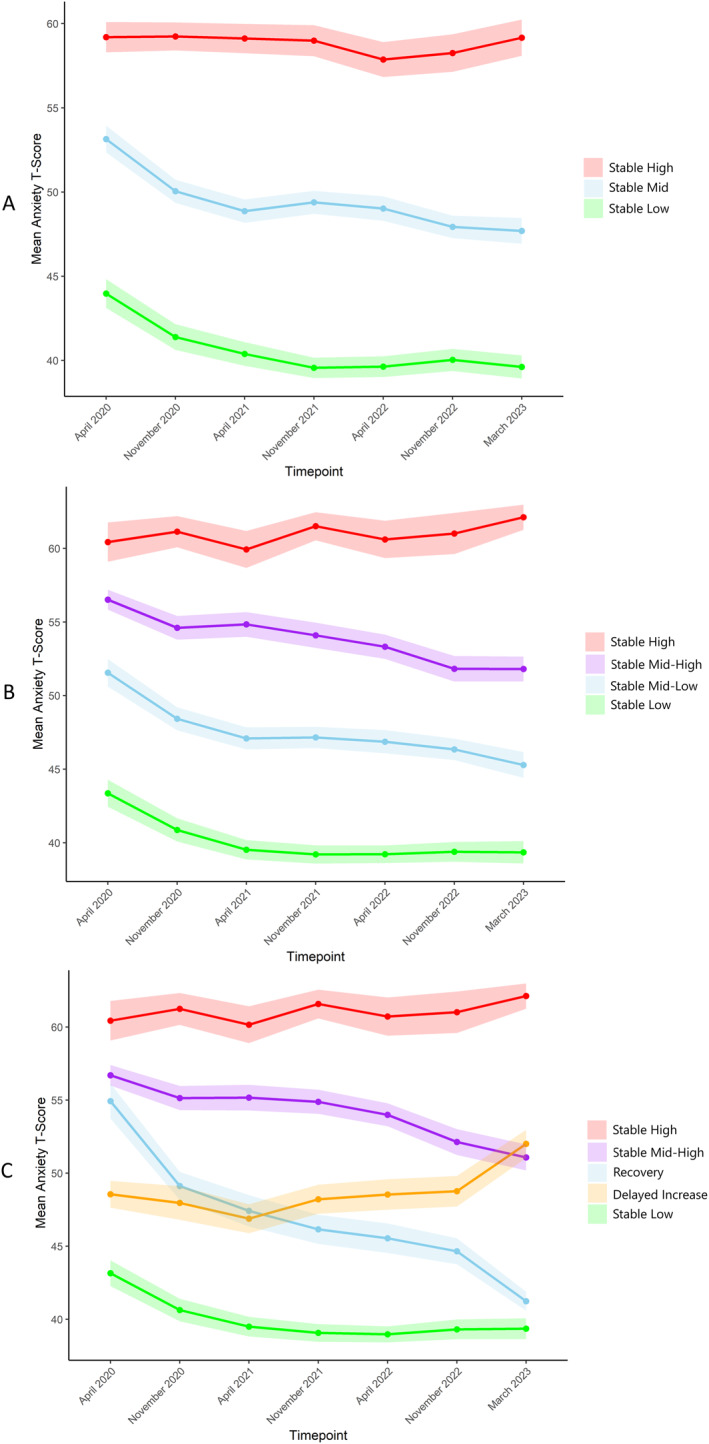
Trajectories of anxiety symptoms in the different clusters of children from April 2020 until March 2023. The shade represents the 95% confidence interval around the mean anxiety in each cluster. Panel A shows the 3‐cluster solution, B the 4‐cluster solution and C the final 5‐cluster solution.

Initially, the k‐means clustering analysis resulted in an optimal clustering (highest Calinski‐Harabasz index) of three longitudinal trajectories; (1) a stable high (*N* = 125, 24.3%), (2) a stable medium (*N* = 214, 41.6%) and (3) a stable low trajectory (*N* = 175, 34.0%). The means (+/− 95% CI) per measurement occasion can be seen in Figure 1 and Table S1. Subsequently, we investigated the four‐cluster solution as it was the next solution with the best fit. The four‐cluster solution contained four stable trajectories; high (*N* = 73, 14.2%), medium‐high (*N* = 122, 23.7%), medium‐low (*N* = 166, 32.3%), low (*N* = 153, 29.8%) (see Figure 1 and Table S1). To investigate possible unstable trajectories, we further explored five clusters in accordance with previous literature on adults and children (de Heer et al., 2025; Pierce et al., 2020; van Loon et al., 2022).

The five‐cluster solution (see Table 2) displayed three stable trajectories; a stable high (*n* = 71, 13.8%), a stable medium (*n* = 109, 21.2%) and a stable low (*n* = 147, 28.6%) trajectory. In addition, two distinct trajectories of interest were identified; (1) a recovering trajectory in which children initially scored high on anxiety, but anxiety decreased during the pandemic and were back to levels of anxiety outside of the (sub)clinical range after the pandemic ended (*n* = 100, 19.5%), and (2) a delayed increase in anxiety as response to the pandemic in which children reported stable anxiety during the pandemic, and an increase in anxiety after the pandemic regulations ended (*n* = 87, 16.9%). The recovery group displays significant recovery throughout, whereas the delayed increase cluster displays a significant increase in anxiety from September 2022 to April 2023 (from 48.8 (47.5–50.0) to 52.0 (50.8–53.3), see Figure 1).

**TABLE 2:** Means of PROMIS anxiety T‐scores by trajectory cluster with 95% confidence intervals (CI) for the 5‐cluster solution.

	5‐cluster solution means (95% CI)				
	Low (*N* = 147, 28.6%)	Medium (*N* = 109, 21.2%)	Recovery (*N* = 100, 19.5%)	Delayed increase (*N* = 87, 16.9%)	High (*N* = 71, 13.8%)
April 2020	43.2 (42.0–44.3)	56.7 (55.7–57.7)	54.9 (53.3–56.6)	48.6 (47.4–49.8)	60.4 (58.5–62.3)
September 2020	40.6 (39.8–41.5)	55.1 (54.2–56.1)	49.1 (48.1–50.2)	48.0 (46.6–49.3)	61.2 (59.9–62.6)
April 2021	39.5 (38.8–40.2)	55.2 (54.2–56.2)	47.4 (46.2–48.6)	46.9 (45.8–48.1)	60.2 (58.7–61.7)
September 2021	39.1 (38.4–39.7)	54.9 (54.0–55.8)	46.2 (45.1–47.2)	48.2 (47.1–49.3)	61.6 (60.5–62.7)
April 2022	39.0 (39.4–39.6)	54.0 (53.1–54.9)	45.5 (44.4–46.7)	48.5 (47.3–49.8)	60.7 (59.1–62.3
September 2022	39.3 (38.5–40.1)	52.1 (51.1–53.2)	44.7 (43.6–45.7)	48.8 (47.5–50.0)	61.0 (59.3–62.7)
April 2023	39.4 (38.5–40.2)	51.1 (49.8–52.3)	41.2 (40.4–42.0)	52.0 (50.8–53.3)	62.1 (61.1–63.2)

*Note*: Pre‐pandemic reference values of the Dutch general population for the mean was 43.8 with cut‐off values for mild (50.8) and severe (61.5) anxiety.

### Risk factors

To identify risk factors for belonging to a certain trajectory of anxiety, we investigated the associations of sociodemographic characteristics. These subgroup analyses found no significant effects of sex, urbanization, education level of the parent, and immigration status of the parents (see Table 3) between the five trajectories.

**TABLE 3:** Sociodemographic characteristics of the different clusters of children and significance of the performed statistical test (either ANOVA or chi‐square tests of equal proportions).

	Low (*N* = 147)	Medium (*N* = 109)	Recovery (*N* = 100)	Delayed increase (*N* = 87)	High (*N* = 71)	Total (*N* = 514)	*p*‐value
Sex; % females	51.7%	54.1%	47.0%	46.0%	57.7%	51.2%	0.998
Age (SD)	13.33 (2.96)	12.55 (2.82)	13.03 (2.99)	13.67 (2.92)	12.64 (2.88)	13.07 (2.94)	0.042
Single parent family; % yes	15.5%	17.0%	20.7%	22.9%	31.4%	20.3%	0.076
Loss of income; % yes	15.0%	25.7%	17.0%	18.4%	59.2%	24.3%	**<0.001**
Urbanization							0.231
>2500 addresses per km^2^	13.4%	21.8%	10.6%	14.1%	19.6%	15.7%	
>1500–2500 addresses per km^2^	19.6%	34.6%	25.8%	32.8%	23.9%	27.1%	
>1000–1500 addresses per km^2^	21.6%	16.7%	25.8%	20.3%	15.2%	20.2%	
>500–1000 addresses per km^2^	27.8%	16.7%	28.8%	17.2%	19.6%	22.5%	
<500 addresses per km^2^	17.5%	10.3%	9.1%	15.6%	21.7%	14.5%	
Education level parent^b^							0.932
Practical vocational	7.7%	5.7%	8.7%	12.0%	7.1%	8.1%	
Medium	44.4%	48.1%	46.7%	43.4%	47.1%	45.8%	
Theoretical vocational	47.9%	46.2%	44.6%	44.6%	45.7%	46.0%	
Parents born in the Netherlands^a^							0.141
Neither (*N*)	90.5%	85.3%	80.0%	88.5%	90.1%	87.0%	
One (*N*)	4.1%	4.6%	11.0%	3.4%	5.6%	5.6%	
Both (*N*)	2.0%	6.4%	1.0%	2.3%	2.8%	2.9%	

*Note*: Bold represents a significant *p*‐value, corrected for multiple testing (*p* < 0.007).

^a^ Percentages may not add up to a 100% as there are missing values.

^b^ Low (primary education, lower vocational education, or lower and middle general secondary education), intermediate (middle vocational education, higher secondary education, or pre‐university education), or high (higher vocational education or university).

The loss of income of parents at any time point was associated with a higher probability of belonging to the stable high anxiety trajectory compared to belonging to any other trajectory (59.2%, OR range pairwise comparisons 4.1–8.2, *p* < 0.001).

## DISCUSSION

In this study we aimed to distinguish different trajectories of anxiety in children during and after the COVID‐19 pandemic (2020–2023). Children followed distinct anxiety trajectories during and after the COVID‐19 pandemic, showing variability in their long‐term mental health responses. We identified five different trajectories of anxiety. Three trajectories showed a stable anxiety trajectory throughout the period, either low anxiety (28.6% of the children), medium anxiety (21.2%) or high anxiety (13.8%). Furthermore, two groups of children showed variability in their trajectories. The first is a recovering trajectory (19.5% of the children), where children initially report high levels of anxiety, but this gradually decreased as the pandemic progressed and returned to normal values after the pandemic period ended. The second is a delayed increase trajectory. These children report higher levels of anxiety after the pandemic and the lockdown measures were abolished. We found that in particular loss of income was strongly associated with a higher probability of belonging to the trajectory of stable high anxiety. The findings suggest that extra financial stressors for parents during the pandemic may contribute to sustained elevated anxiety levels in children and adolescents.

The trajectories in the current study were similar to previous findings in both children and adults. Most studies report stable low, medium and high trajectories of mental health, that is, depressive symptoms or anxiety symptoms (de Heer et al., 2025; Guzman Holst et al., 2023; Houghton et al., 2022; Kaman et al., 2024; van Loon et al., 2022), which is common when using a clustering algorithm as it maximizes distance between clusters. Regarding these stable trajectories, the stable low trajectory shows that around a quarter of all children are resilient to the effects of the pandemic on their levels of anxiety. Their scores were not elevated compared to the pre‐pandemic reference data.

In contrast to previous COVID‐19 studies, we followed children until after the pandemic (April 2023). A recovering trajectory has also been observed in earlier studies on child mental health (de Heer et al., 2025), although these studies often covered shorter time periods, limiting the extent to which full recovery could be detected. The children in our recovering trajectory showed a complete return to anxiety levels within the normal range following the pandemic.

Alongside this recovering group, our study also revealed a different pattern: a delayed increase trajectory, characterized by an increase in anxiety symptoms after the pandemic had ended. These particular findings highlight the importance of extending mental health monitoring beyond the crisis phase and suggest that long‐term follow‐up is essential to fully understand the enduring effects of the pandemic, on child mental health. It could be possible that this delayed increase is caused by other events outside of the pandemic—qualitative research is necessary to investigate this further.

The delayed increase to the pandemic may represent a subgroup of the population that benefited from the pandemic regulations (Bruining et al., 2021). In earlier research some children as well as adults adapted faster to pandemic regulations and may have even preferred them. Another possibility is that this group had trouble returning to society and that the pressures of participating (socially, academically) increased symptoms of (social) anxiety (Thompson et al., 2021). This is supported by our finding that the average age of this cluster was slightly higher than in the other clusters. Relationships with peers and participation with society may be more anxiety‐inducing to older children as they may feel they have to meet certain expectations. Further studies are required to assess this effect of returning to normal mental health after the pandemic and what separates children who seem to have thrived (with respect to mental health) during the pandemic from the others.

Compared to studies on the natural development of anxiety during childhood and adolescence (i.e., without the experience of a global stressor) there is overlap with natural occurring trajectories of anxiety (Broeren et al., 2013; Letcher et al., 2012). Specifically, the stable low, medium and high trajectories of anxiety occur naturally throughout childhood in most studies that have attempted to identify anxiety trajectories (Weems, 2008). Certain studies have also found a decreasing or increasing trajectory of anxiety, though these studies are sparser and results have been inconsistent (Weems, 2008). Therefore, it is important to take into account that the trajectories we found in this study could have occurred without a pandemic, though the proportion of children belonging to the recovering and delayed increase trajectories in our study are much larger (17%–20%) than generally found in studies prior to the pandemic (where recovery groups only accounted for 5%–10% of the samples (Weems, 2008)).

The second aim in this study was to identify risk factors for belonging to different trajectories of anxiety during and after the pandemic. Only one risk factor emerged: parental loss of income due to the pandemic. Previous research has shown that loss of income is a risk factor for worse mental health of children during the pandemic (Guzman Holst et al., 2023; Hafstad et al., 2021; Raw et al., 2021). The loss of income of parents is likely to increase parental stress (McArthur et al., 2023), which has also been found to decrease mental health (specifically anxiety and depression) in their children (Guzman Holst et al., 2023; Ravens‐Sieberer et al., 2022; Raw et al., 2021). Previous studies have identified that family composition (such as being from a single parent family) is an important risk factor for more child mental health problems during the pandemic (Hafstad et al., 2021; Luijten et al., 2021; Ravens‐Sieberer et al., 2022). Although parental emotional functioning and family dynamics are likely important contributors to trajectories of child anxiety (Pagani et al., 2008), we could not assess these in the current study. Future research is needed to better understand how parental stress and family context shape children's mental health during crises.

In contrast to previous studies, we did not find that sex is a risk factor of belonging to any specific trajectory. Previous research has shown that adolescent girls generally report higher levels of anxiety and mental health complaints (Hartas, 2024). It could be that within each trajectory, adolescent girls report higher anxiety levels than boys, but sex has no effect on the shape of the trajectories over time (Houghton et al., 2022). As such, our findings do not necessarily contradict earlier studies but suggest that while sex may relate to overall anxiety levels, it does not predict a specific negative or positive trajectory of anxiety.

### Strengths and limitations

An important strength of our study is that we followed a large group of children, representative of the Dutch population, for a longer period of time, allowing us to include post‐pandemic measurement occasions. The inclusion of these measurement occasions has provided new insights into the effect of “returning to normal” on the mental health of children. We measured anxiety consistently in the same months (i.e., April/May and October/November) to prevent seasonal effects from playing a (substantial) role in differences in anxiety scores.

Furthermore, we used the validated self‐report PROMIS questionnaire to assess anxiety, which has previously shown to have strong psychometric properties and a broad range, suitable for a general population (Klaufus et al., 2021). Previous studies often rely on measures of anxiety more suitable for clinical populations and screening of psychiatric disorders (such as the Strengths and Difficulty Questionnaires or the proxy‐reported Brief Problem Monitor), which are less suitable for the purpose of monitoring change in general populations. However, the self‐reported scores may be influenced by reporting biases (e.g., social desirability or differences in interpretation), which should be considered when interpreting the findings.

A limitation of the current study is the lack of pre‐pandemic measures, which prevents us from modeling the change from the pre‐pandemic to the pandemic period. As a result, we cannot determine whether children in the recovery group returned to their individual pre‐pandemic levels of anxiety and whether the other groups showed a stable or changing trajectory from before to during the pandemic. However, previous cross‐sectional research in a comparable representative Dutch sample suggests that average pre‐pandemic anxiety scores closely resemble the scores observed in our recovery group at the final measurement (Luijten et al., 2021; van Oers et al., 2024; Zijlmans et al., 2025). Moreover, studies that have included pre‐pandemic measures have also been able to identify a recovery trajectory (de Heer et al., 2025), thus it is likely that these children did recover to their pre‐pandemic anxiety levels. In addition, there could be selection bias as participants were only included if they had completed multiple (>3) measurement occasions, but children that report worse outcomes may be more likely to drop‐out. This would mainly impact the group sizes of each trajectory and not necessarily the shape of the trajectories.

Another limitation is that our longitudinal sample differed from the representative sample of the general population on loss of income from the parents. Considering that this is one of the main risk factors for belonging to an unfavorable trajectory of anxiety, it is possible that the group sizes for these trajectories found in our sample may be overrepresented. In this study we had no additional information on (teletherapy) treatment for mental health during the pandemic and therefore cannot fully exclude effects of treatment (e.g., more children in the recovery trajectory could be receiving mental health treatment than in others trajectories). It is also important to emphasize that our sociodemographic characteristics were self‐reported and may not be entirely accurate. However, given the larger sample size this likely did not impact the overall effects found in this study.

## CONCLUSION

In this study we identified five distinct trajectories of anxiety during and after the pandemic. A non‐negligible group of children showed increased anxiety levels after the pandemic regulations were abolished, highlighting that the pandemic may have a long‐term impact. Children from families experiencing parental income loss were at greater risk of following a less favorable trajectory. We show the importance of longer‐term monitoring of children after a global stressor. This can be done on a population‐level through longitudinal epidemiological studies (such as the Netherlands Twin Register) without increasing burden on mental health care services. By monitoring we can identify children who require help from mental healthcare professionals and forward them, so that these children recover rather than remaining in a state of stable, high anxiety. Overall, child mental health in the post‐pandemic landscape maintains a relevant and important topic that should be taken into account when developing future policies on pandemic preparedness and healthcare.

## AUTHOR CONTRIBUTIONS

**Michiel A. J. Luijten.** Conceptualization (equal); formal analysis (lead); methodology (lead); visualization (lead); writing—original draft (lead); writing—review and editing (equal). **Hedy A. van Oers.** Data Curation (lead); writing—review and editing (equal). **Jacintha M. Tieskens.** Conceptualization (supporting); writing—review and editing (equal); **Marlene A. Werner.** Conceptualization (supporting); writing—review and editing (equal). **Josjan Zijlmans.** Conceptualization (supporting); writing—review and editing (equal). **Lotte Haverman.** Conceptualization (equal); writing—review & editing (equal); supervision (supporting). **Tinca J. C. Polderman.** Conceptualization (equal); writing—review and editing (equal); supervision (equal); funding acquisition (lead). **Lianne P. de Vries.** Conceptualization (equal); writing—original draft preparation (supporting); writing—review and editing (equal); supervision (equal).

## CONFLICT OF INTEREST STATEMENT

The authors have declared that they have no competing or potential conflicts of interest.

## ETHICAL CONSIDERATIONS

All participants and their parents provided informed consent. Data collection for the study was approved by the Medical Ethics Testing Committee (METC) of the Amsterdam UMC (W20_132 # 22.444), 31st of October 2022. The study was conducted in line with the ethical standards stated in the 1964 Declaration of Helsinki and its later amendments.

## Supporting information

Table S1

## Data Availability

The data that support the findings of this study are available from the corresponding author upon reasonable request.
