# Supplementary material for: The pandemic generation: Investigating the long‐term impact of a large‐scale stressor on the anxiety of children
Source: JCPP Adv. 2026 Feb 25:e70105. Online ahead of print. doi: 10.1002/jcv2.70105 (PMC13339612; doi:10.1002/jcv2.70105)
Supplement: Supplementary file 1 — Table S1 [file JCV2-9999-e70105-s001.docx]

**The pandemic generation: Investigating the long-term impact of a large-scale stressor on the anxiety of children**

**Supporting Information**

| **Table S1.** Means of PROMIS Anxiety T-scores by trajectory cluster with 95% confidence intervals (CI) for the 3-cluster and 4-cluster solutions. | | | | | | | | |  |
| --- | --- | --- | --- | --- | --- | --- | --- | --- | --- |
|  | **3-cluster solution means (95% CI)** | | |  | **4-cluster solution means (95% CI)** | | | |  |
|  | **High  (N = 125)** | **Medium  (N = 214)** | **Low  (N = 175)** |  | **High  (N = 73)** | **Medium-High  (N =122)** | **Medium-Low  (N = 166)** | **Low  (N = 153)** |  |
| **April 2020** | 59.2 (58.0 – 60.4) | 53.1 (52.1 – 54.2) | 44.0 (42.9 – 45.1) |  | 60.4  (58.5 – 52.3) | 56.5  (55.6 – 57.4) | 51.5  (50.3 – 52.8) | 43.4  (42.2 – 44.6) |  |
| **September 2020** | 59.2  (58.3 – 60.2) | 50.0  (49.3 – 50.8) | 41.4  (40.5 – 42.2) |  | 61.1  (59.9 – 62.4) | 54.6  (53.7 – 55.5) | 48.4  (47.5 – 49.3) | 40.9  (40.0 – 41.8) |  |
| **April 2021** | 59,1 (58.1 – 60.1) | 48.9  (48.1 – 49.7) | 40.4  (39.7 – 41.1) |  | 59.9  (58.4 – 61.4) | 54.8  (53.9 – 55.8) | 47.1  (46.2 – 47.9) | 39.5  (38.8 – 40.2) |  |
| **September 2021** | 58.9 (58.0 – 60.0) | 49.4 (48.7 – 50.1) | 39.6 (38.9 – 40.2) |  | 61.5  (60.4 – 62.6) | 54.1  (53.2 – 55.0) | 47.2  (46.4 – 47.9) | 39.2  (38.5 – 39.9) |  |
| **April 2022** | 57.9 (56.7 – 59.0) | 49.0 (48.2 – 49.9) | 39.6 (38.9 – 40.3) |  | 60.6  (59.1 – 62.1) | 53.3  (52.4 – 54.3) | 46.9  (46.0 - 47.8) | 39.2  (38.6 – 39.9) |  |
| **September 2022** | 58.3 (56.9 – 59.6) | 47.9 (47.1 – 48.7) | 40.0  (39.3 – 40.8) |  | 61.0  (59.2 – 62.7) | 51.8  (50.8 – 52.8) | 46.3  (45.5 – 47.2) | 39.4  (38.6 – 40.2) |  |
| **April 2023** | 59.2 (57.8 – 60.6) | 47.7 (46.7 – 48.7) | 39.6 (38.8 – 40.4) |  | 62.1  (61.1 – 63.2) | 51.8  (50.6 – 53.0) | 45.3  (44.2 – 46.4) | 39.4  (38.5 – 40.3) |  |
